# Increased Immunohistochemical Expression of Stimulator of Interferon Genes (STING) in Renal Cancer with Venous Tumor Thrombus Is Associated with Worse Prognosis

**DOI:** 10.3390/biomedicines13112674

**Published:** 2025-10-30

**Authors:** Sumit Sharma, Michał Kunc, Rafał Pęksa, Aleksandra Ciarka, Weronika Łyzińska, Le Qu, Piotr Radziszewski, Łukasz Zapała

**Affiliations:** 1 Clinic of General, Oncological and Functional Urology, Medical University of Warsaw, Lindleya 4, 02-005 Warsaw, Poland; piotr.radziszewski@wum.edu.pl; 2 Department of Pathomorphology, Medical University of Gdansk, 80-214 Gdańsk, Poland; michal.kunc@gumed.edu.pl (M.K.); rafal.peksa@gumed.edu.pl (R.P.); aleksandra.ciarka@gumed.edu.pl (A.C.); 3 Scientific Circle of Pathomorphology, Medical University of Gdańsk, 80-214 Gdańsk, Poland; weronika.lyzinska@gumed.edu.pl; 4 Department of Urology, Jinling Hospital, Affiliated Hospital of Medical School, Nanjing University, Nanjing 210000, China; septsoul@hotmail.com

**Keywords:** renal cell carcinoma, renal tumor thrombus, tumor microenvironment, STING

## Abstract

**Background/Objectives**: We focused on the expression of a novel immune marker, cytoplasmic stimulator of interferon genes (STING), in the cohort of primary renal cell cancer (RCC) with venous tumor thrombus (VTT), in conjunction with the assessment of tumor-infiltrating leucocytes (TILs). **Methods**: The study group comprised 82 patients with clear cell RCC and VTT, operated on in the years 2012–2019 in two university urological centers. Tissue microarrays were constructed, and respective antibodies were used for staining purposes. The biomarkers were analyzed in primary RCC and VTT. **Results**: The frequency of STING expression in both analyzed compartments was similar (*p* = 0.18). Its presence correlated with no clinicopathological features but for necrosis in VTT only (*p* = 0.0023). PD-L1 expression in the primary tumor was associated with STING in tumor cells in the same compartment (*p* = 0.02). On the contrary, VISTA expression was correlated with the presence of STING in VTT. TIL presence was associated with positive PD-L1 (*p* = 0.008) and STING (*p* < 0.05) expression in the primary tumor. Strong STING expression in VTT was associated with inferior overall survival (OS) (*p* = 0.0061). TIL presence emerged as a robust prognostic factor for OS in both primary tumor (*p* = 0.021) and VTT (*p* = 0.034). **Conclusions**: We presented for the first time the prognostic values of STING in a contemporary cohort of RCC patients with VTT. STING expression in VTT showed prognostic potential, while TIL assessment proved to be a particularly valuable prognostic tool that can be readily implemented in routine pathological evaluation.

## 1. Introduction

Nowadays, there is a growing interest in identifying novel predictive and prognostic biomarkers for renal cell carcinoma (RCC) [1]. As RCC represents a truly heterogeneous disease, trends towards personalization of the treatment based on individuals’ cancer profiles may become the pillars of future immunotherapy concepts. In this setting, cases with coexisting venous tumor thrombus (VTT) pose not only a challenge for the clinicians but may require completely different systemic management [2].

The cytoplasmic stimulator of interferon genes (STING) pathway serves as a key activator of the immune response to intracellular double-stranded DNA fragments [3,4]. Its stimulation triggers upregulation of several inflammatory genes, such as tumor necrosis factor (TNF), or interferons α and β (IFN α and β) [3,5]. Corrales et al. found that intratumoral injections of STING agonists initiated regression of established tumors in mice via IFN-dependent pathways [6]. Moreover, upregulation of STING resulted in the promotion of chemokines, such as CXCL9-11 and CCL5, facilitating T cell trafficking to the primary tumor and its microenvironment [6,7]. Additionally, STING can reverse immunosuppressive conditions by reprogramming myeloid-derived suppressor cells into antigen-presenting cells [8,9] and polarizing tumor-promoting M2 macrophages toward the anti-tumor M1 phenotype [10].

At the early stages, STING activation ceases the onset of tumor, while chronic stimulation of the STING pathway can promote inflammation-driven carcinogenesis. This dual nature of STING, as both a potential tumor suppressor and promoter, highlights the complexity of its role in cancer biology. While STING expression has been predominantly studied in T lymphocytes and macrophages [5], its role in tumor microenvironments varies across malignancies. In colon adenocarcinoma and breast cancer, STING functions as a tumor suppressor [11,12]. However, limited studies examined STING expression in RCC subtypes, including MiT family translocation RCCs [13], fumarate hydratase-deficient RCC [14], or medullary RCC [15], though sample sizes were relatively small.

Interestingly, the assessment of tumor-infiltrating immune cells (TAICs) or lymphocytes (TILs) has emerged as a valuable prognostic tool in various malignancies, particularly breast cancer, in which it can even aid estimation of the effects of planned chemotherapy and immunotherapy [16]. Preliminary reports raised this issue in RCC, emphasizing the possible link between the subpopulations of TAICs and clinicopathological characteristics, though this remains an area requiring further investigation [17].

We have previously characterized immune biomarkers in RCC patients with VTT, including immune checkpoint molecules (PD-L1 and VISTA), and demonstrated their compartment-specific expression patterns and prognostic significance [18]. The present study aimed to evaluate STING expression in both RCC and VTT compartments and assess its prognostic value in conjunction with TIL analysis. We hypothesized that STING expression patterns would differ between compartments and provide prognostic information in this challenging RCC presentation.

## 2. Materials and Methods

### 2.1. The Cohort

We analyzed consecutive patients diagnosed with clear cell renal carcinoma (ccRCC) with concomitant venous tumor thrombus staged cT3a or higher, in whom either nephrectomy with or without cavotomy and thrombectomy was performed. The cohort was treated in the years 2012–2019 in the two academic urological departments. The study group also comprised cN1 cases, in whom lymph node dissection was performed as well, while metastatic cases were treated with a surgical approach upfront, either with an attempt at complete resection of all tumor lesions or as a cytoreductive nephrectomy. The utilized approaches were lumbotomy or laparotomy. No neoadjuvant treatment was administered in any of the individuals enrolled. Clinical data were collected from the local hospital in- and out-patients’ registries, including tumor advancement assessment based on CT or MRI imaging (2017 TNM classification system [19]) and pathological analyses (based on WHO/ISUP classifications), and were supplemented with telemedicine patients’ consultations, if needed for the purpose of the details of the follow-up and further statistical analyses. The scoring systems used for the evaluation of sarcomatoid/rhabdoid features and tumor necrosis were binary (present vs. absent). Patients (*n* = 8) with incomplete clinicopathological data or survival data were excluded from further analysis. Informed consent was collected from all the patients included in the study. The study was carried out after the agreement granted by the local ethics committee, vote No. AKBE/72/2021 (Medical University of Warsaw).

### 2.2. Tissue Microarrays

Tissue microarrays (TMAs) with representative samples of matched primary renal tumor and venous tumor thrombus were constructed, as described elsewhere, with manual Tissue Arrayer MTA-1 (Beecher Instruments, Inc., Sun Prairie, WI, USA) using 1.5 mm core needles [20]. The respective *n* = 3 cores (one from the central and two from the peripheral areas) from both VTT and the main primary lesion were sampled, as described previously [20]. Then, the prepared sections were stained with anti-STING antibody (OTI4E12; product #MA5-26032, dilution at 1:100, ThermoFisher Scientific, Waltham, MA, USA). PD-L1 and VISTA expression were evaluated, as reported previously [18,21]. Respective negative (liver samples) and positive controls (tonsil, placenta) were included in TMAs [21]. The stainings were assessed by 2 pathologists experienced in uropathology (MK and RP). The expressions of the above-mentioned markers were assessed in tumor cells in two different compartments, i.e., in the tumor mass and tumor thrombus. Cytoplasmic and membranous STING expression in tumor cells (TCs) was assessed by calculating an H-score (ranging from 0 to 300), derived from multiplying the percentage of positive cells (0–100%) by the staining intensity (scored as 0, 1+, 2+, or 3+), as proposed previously [22]. Cytoplasmic staining of tumor cells was quantified, with immune and endothelial cells serving as internal positive controls. The STING expression cutoff of H-score > 100 (high vs. low) was used, as it was noted to be a 75-percentile value for STING expression in the VTT compartment. Moreover, the presence of tumor-infiltrating lymphocytes (TILs) was assessed dichotomously as present (at least 1% of stromal TILs) or absent (less than 1% of stromal TILs). The 1% cutoff was implemented due to practical reasons, as it dichotomously differentiates tumors with no lymphocytes and those with at least some lymphocytic infiltration, resulting in satisfactory reproducibility.

### 2.3. Statistics

Overall survival (OS) was determined as the interval between diagnosis and death due to any cause. For statistical purposes Statistica 13 (RRID:SCR_014213, Tibco, Palo Alto, CA, USA; licensed to Medical University of Gdańsk) and the R statistical environment (R Core Team: Vienna, Austria) were utilized [23]. Continuous and categorical variables were compared using the Wilcoxon rank-sum test and Chi-square test, respectively. Univariate survival analysis was conducted with Kaplan–Meier curves and compared using the log-rank test. Multivariate survival analysis was performed using Cox proportional hazards regression and Variance Inflation Factor (VIF) analysis of collinearity. Data visualization was carried out with the ggplot2 and survminer packages in R [24]. A *p*-value ≤ 0.05 was considered statistically significant.

## 3. Results

### 3.1. Patients

The studied cohort comprised 82 patients (female *n* = 37, male *n* = 45) with a median age of 66 (age range 60–72). The basic pathological features are presented in Table 1. Of note, these were mainly T3a cases, but in 27% N+ and in 28% M+ cases were observed, respectively. Mean follow-up was 35.3 months (median 27, range 1–109), and 3-year overall survival reached 70%.

### 3.2. Cytoplasmic Expression of STING on Tumor Cells and Its Correlations with Pathological Features

**Figure 1:**
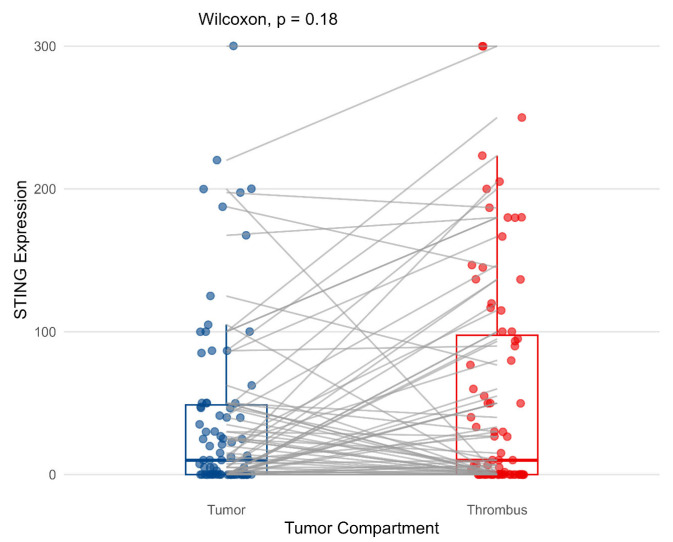
Cytoplasmic expression of STING in primary tumor and venous tumor thrombus. The associations were estimated with the Wilcoxon test.

**Figure 2:**
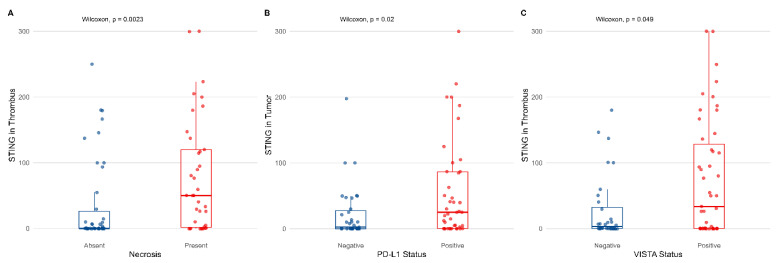
Cytoplasmic expression of STING in venous tumor thrombus (**A**,**C**) or primary tumor (**B**) according to necrosis (*p* < 0.01), PD-L1 (*p* < 0.05), and VISTA (*p* < 0.05). The associations were estimated with the Wilcoxon test.

The frequency of STING in both the analyzed compartments was similar, with no statistically significant differences noted for the membranous and cytoplasmic staining (*p* = 0.18, Wilcoxon test, Figure 1). Then, cytoplasmic STING expression was not significantly associated with most clinicopathological variables (tumor diameter, presence of nodal or distant metastases, and tumor grade) when analyzed in both primary tumor and VTT (please refer to Table S1 in the Supplementary Materials). However, STING expression in VTT was positively correlated with necrosis (*p* = 0.0023, Wilcoxon test, Figure 2A).

**Figure 3:**
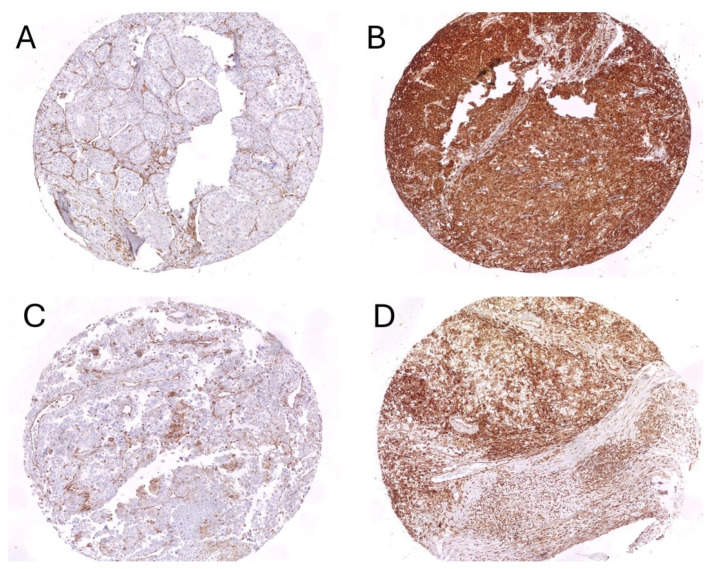
Representative examples of STING staining in tumor (**A**,**B**) and thrombus (**C**,**D**). Negative reaction in tumor cells with positive control in immune and endothelial cells (**A**,**C**) and strong diffuse positive staining in tumor cells (**B**,**D**).

Median H-score values for STING in tumor thrombus were 10, and for STING in tumor—10, as well. Representative examples of STING staining in primary RCC and venous tumor thrombus were presented in Figure 3.

### 3.3. Association Between STING Expression and Markers of Inflammatory Response

As for immune checkpoint inhibitors, PD-L1 in the primary tumor was positively correlated with STING in TCs in the same compartment (*p* = 0.02, Wilcoxon test), but not in the tumor thrombus (Figure 2B). On the contrary, VISTA expression was associated with the presence of cytoplasmic STING in VTT (*p* = 0.049, Wilcoxon test, Figure 2C).

### 3.4. Association Between TILs and Clinicopathological Variables in Different Compartments

**Figure 4:**
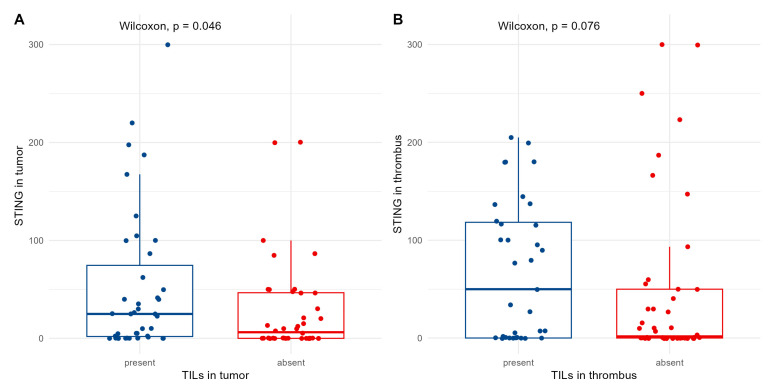
Cytoplasmic expression of STING and presence of TILs in primary tumor (**A**) and venous tumor thrombus (**B**).

TILs were present in 39 primary tumors (47.6%) and 35 venous thrombi (42.7%). Their presence was associated with positive PD-L1 expression in the tumor (*p* = 0.008, Chi-square). Moreover, we observed a positive correlation between the STING expression and TIL presence in the primary tumor only (Figure 4A,B) (*p* < 0.05). No statistically significant correlations with other clinicopathological variables were observed.

### 3.5. The Prognostic Value of STING and TILs in the Studied Cohort

**Figure 5:**
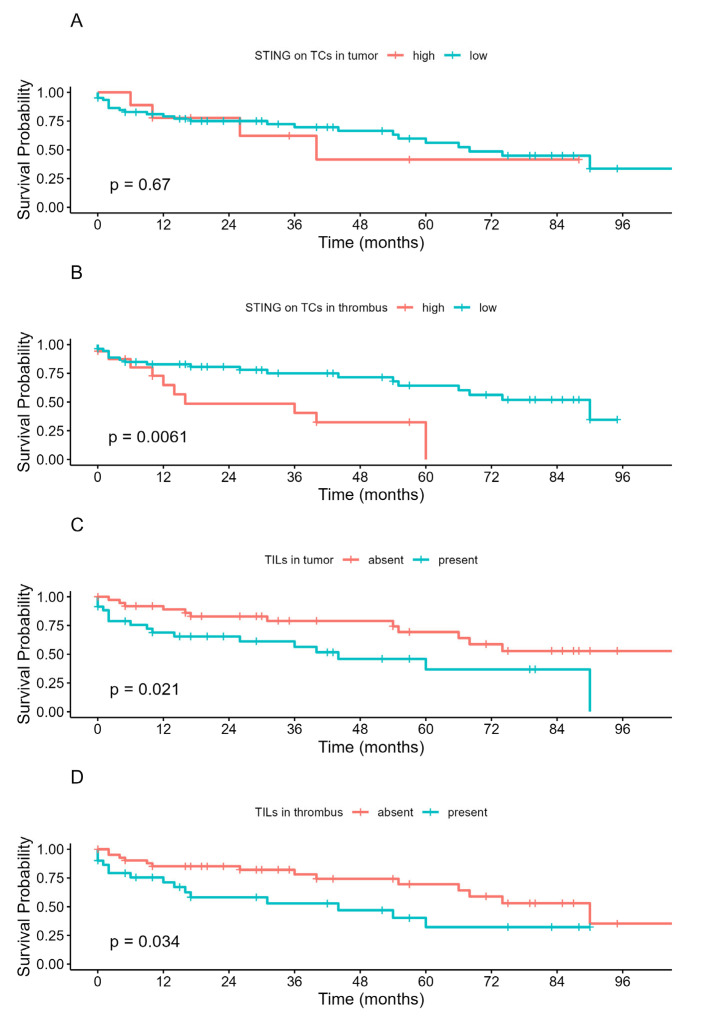
Kaplan–Meier curves for overall survival according to cytoplasmic STING expression on tumor cells in the tumor mass (**A**), and venous tumor thrombus (**B**), and the presence of TILs in both compartments analyzed separately (**C**,**D**). *p*-values were calculated with the log-rank test. Abbreviations: low—negative expression; high—positive expression.

**Figure 6:**
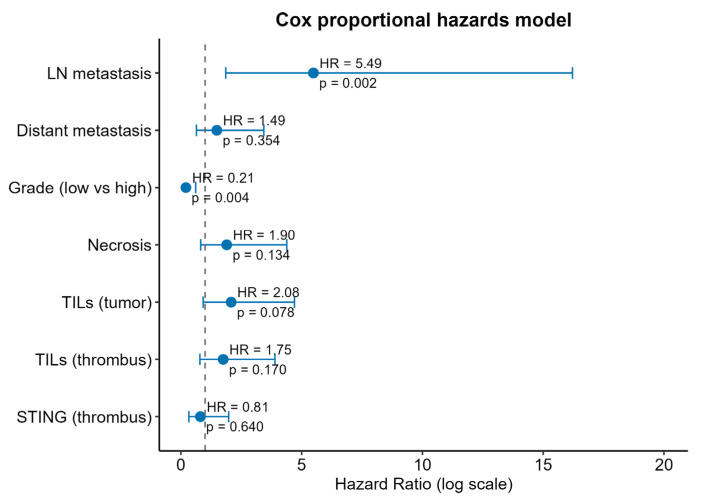
Cox proportional hazards model for prediction of overall survival. Abbreviations: LN—lymph nodes; TILs—tumor-infiltrating leucocytes; STING—stimulator of interferon genes; HR—hazard ratio.

Then, we focused on the survival differences determined by the STING expression and the presence of TILs. STING expression in primary tumor cells was not associated with any differences in OS (Figure 5A). On the other hand, strong positive STING expression (H-score > 100) in VTT was associated with inferior OS (*p* = 0.0061) (Figure 5B). Finally, the presence of TILs proved to be a prognostic factor of OS when analyzed in both primary tumor (*p* = 0.021) and venous tumor thrombus (*p* = 0.034, Figure 5C,D). However, in multivariate analysis, only tumor grade and nodal metastases retained statistical significance (Figure 6), with low multicollinearity found in regression models (please refer to Supplementary Materials, Table S2).

## 4. Discussion

This study presents the first analysis of STING expression pattern and its prognostic value in the contemporary cohort of RCC patients with VTT. We demonstrated compartment-specific differences in STING correlations with respective clinicopathological features and identified TILs as a promising prognostic marker. These findings provide insights into the distinct tumor microenvironments of primary RCC and VTT, with potential implications for treatment stratification.

The dual perspective of STING function, as far as the anticancer response is considered, exists with context-dependent pro- and anti-tumor effects [25]. In a mouse model of adenocarcinoma, Hu et al. demonstrated that systemic administration of STING agonists eradicates dormant metastasis, being a specific checkpoint against the progression [26]. Interestingly, the authors reported that upregulation of STING may decrease sensitivity to genotoxic treatment in breast cancer models [4]. As a further example, Chen et al. observed that cancer cells may activate STING signaling via cGAMP transfer and, in turn, produce the inflammatory cytokines, i.e., IFN-α and TNF-α [27], while other authors reported that STING activation may upregulate immunosuppressive cytokines, such as IL-10 and subsequently activate inhibitory regulatory T cells [28]. Interestingly, it was reported that STING is highly expressed in some cases of RCC [25,29] and may be associated conversely with TIL presence and poor prognosis [22,29]. Our findings partially align with this observation, as STING expression in VTT correlated with inferior overall survival, while primary tumor STING expression showed no prognostic significance. This compartment-specific difference suggests distinct biological processes governing STING function in different tumor sites. The correlation between STING expression and necrosis observed exclusively in the VTT compartment supports previous findings by Marletta et al., who hypothesized that elevated STING is a marker of aggressive RCC [22]. They proposed it to be the future biomarker candidate of great prognostic value, especially when combined with tumor size, grading, and necrosis [22]. In RCC specifically, STING appears to promote tumor growth through innate immune processes, including mitochondrial reactive oxygen species (ROS) maintenance [30]. This association may reflect oxidative stress-induced cellular damage, which could be more pronounced in the hypoxic VTT environment compared to primary tumors with established vascular supply [31].

Therapeutic approaches targeting STING have shown promise, with Nakamura et al. demonstrating preliminary findings on STING agonist-loaded lipid nanoparticles (STING-LNPs) in the treatment of lung metastatic RCC [32]. Together with the assessment of tumor inflammatory subpopulations, STING was also found to be a prognostic tool significantly associated with tumor necrosis, sarcomatoid dedifferentiation, and distant metastasis in RCC [22]. Hence, the STING pathway may shape tumor microenvironment via multimodal proinflammatory axes, leading to tumor growth and metastases on one hand, and the necessity to implement multidrug therapies in conjunction with STING agonist on the other. Therefore, STING targeting only was proved to have minimal anticancer efficacy in clinical trials [25,33]. It was found that activation of STING may reinforce the expression of checkpoint ligands, as well [34]. In the present study, we found interesting correlations between STING and immune checkpoint markers in different compartments of RCC with VTT. Thus, PD-L1 in the primary tumor was associated with the positive STING in TCs in the same compartment, while VISTA expression was correlated with the STING staining in VTT. STING activation can upregulate checkpoint ligand expression, with Fu et al. demonstrating that STING stimulation with cyclic dinucleotides resulted in anti-tumor activity and PD-L1 upregulation, possibly through NF-κB signaling [35,36]. The combination of STING agonists with PD-1 blockade may overcome resistance to immune checkpoint monotherapy, supporting the rationale for combination therapeutic approaches [35].

Although the observation of TIL presence in RCC was reported, its value in the assessment of prognosis remains uncertain. TIL assessment emerged as a particularly valuable finding in our study, showing prognostic significance in both compartments. Unlike STING immunohistochemistry, TIL evaluation requires no additional staining and can be readily incorporated into routine pathological assessment. This practical advantage, combined with its promising prognostic value, makes TIL assessment an attractive biomarker for clinical implementation. Previous studies examining TIL subpopulations in RCC showed variable correlations with STING expression. It is worth emphasizing that possible interactions with TIL presence and immune checkpoint markers may exist, as in RCC tumors with PD-1-positive TILs are thought to possess more aggressive clinical characteristics [37]. Here, we found that TILs were associated with positive PD-L1 and STING expression in the tumor compartment only. Some authors focused on the analyses of subpopulations, indicating that there was no correlation between STING and elevated levels of CD8+ T cells in the tumor infiltrates [22]. Xu et al. reported on the link between the M1 macrophage subpopulation together with neutrophils and basic tumor characteristics, i.e., staging and grading (the increased M1 and decreased neutrophil levels from G1 to G4 grading) [17]. The authors also observed that M0 cases and N+ patients were found to have increased M1 levels. Furthermore, these were mainly the M1 fraction and neutrophils that were significantly associated with OS in RCC individuals (*n* = 533 cases diagnosed with clear RCC recorded in Cancer Genome Atlas, in years 2004–2015) [17]. Then, some latest findings impose the idea of modification of the subpopulations of TILs via the exposition to hypoxic conditions, which, in turn, enhances effector functions, leading to formation of tissue-resident memory-like CD8+ T cells [38].

Several limitations of the study warrant consideration. The retrospective design introduces potential selection bias. The use of tissue microarrays, while standardized, may not capture intratumoral heterogeneity present in whole tissue sections. The absence of TIL subpopulation analysis limits mechanistic insights. Additionally, the loss of STING prognostic significance in multivariate analysis suggests that its clinical utility may be limited compared to established prognostic factors. Future studies should include larger cohorts, validation in independent datasets, and functional analyses to clarify the mechanistic basis for compartment-specific STING behavior.

## 5. Conclusions

This study demonstrates compartment-specific STING expression patterns in RCC patients with VTT, with prognostic significance limited to the VTT compartment in univariate analysis. However, TIL assessment emerged as a more robust prognostic tool, shaping the immune response to RCC with VTT. The practical advantage of TIL evaluation, requiring no additional staining, combined with its prognostic reliability, supports its potential implementation in routine pathological practice. The distinct correlations between STING and immune checkpoint markers in different compartments suggest complex immune regulatory mechanisms that warrant further investigation. While STING represents an interesting research target, TIL assessment appears more immediately applicable for clinical prognostication in RCC patients with VTT.

## Figures and Tables

**Table 1:** Basic pathological features of the studied group.

Feature		*n* (Percentage,%)
Grade	G2-G3	55 (67)
	G4	27 (33)
Tumor	3a	79 (96.5)
	3b	1 (1.5)
	3c	-
	4	1 (1.5)
Nodes	0	67 (73)
	1	15 (27)
Metastases	0	59 (72)
	1	23 (28)
Sarcomatoid features	absent	76 (93)
	present	6 (7)
Rhabdoid features	present	73 (89)
	absent	9 (11)
Tumor necrosis	present	38 (46)
	absent	44 (54)

## Data Availability

The original contributions presented in the study are included in the article/Supplementary Material, further inquiries can be directed to the corresponding authors.

## Supplementary Materials

The following supporting information can be downloaded at https://www.mdpi.com/article/10.3390/biomedicines13112674/s1. Table S1: Cytoplasmatic expression of STING on tumor cells and its correlations with pathological features. The associations were estimated with the Wilcoxon test; Table S2: VIF analysis of collinearity of the clinicopathological data. Abbreviations: LN—lymph nodes, VTT—venous tumor thrombus, VIF—Variance Inflation Factor.
