# Supplementary material for: Increased Immunohistochemical Expression of Stimulator of Interferon Genes (STING) in Renal Cancer with Venous Tumor Thrombus Is Associated with Worse Prognosis
Source: Biomedicines. 2025 Oct 30;13(11):2674. doi: 10.3390/biomedicines13112674 (PMC12650444; doi:10.3390/biomedicines13112674)
Supplement: Supplementary file 1 [file biomedicines-13-02674-s001.zip › biomedicines-3915609-supplementary.pdf]

biomedicines

MDPI

Table S1. Cytoplasmatic expression of STING on tumor cells and its correlations with pathological features. The associations were estimated with the Wilcoxon test. Abbreviations: VTT—venous tumor thrombus.

Clinicopathological variables	Cytoplasmatic expression of STING	
	**Primary tumor**	**VTT**
nodal status	$p = 0.74$	$p = 0.4$
distant metastases	$p = 0.13$	$p = 0.064$
grading	$p = 0.7$	$p = 0.1$
necrosis	$p = 0.26$	$p = 0.0023$

Table S2. VIF analysis of collinearity of the clinicopathological data. Abbreviations: LN—lymph nodes, VTT—venous tumor thrombus, VIF—Variance Inflation Factor.

**Variable**	**VIF**	**Interpretation**
LN metastases	1.45	Low collinearity
distant metastases	1.098	Low collinearity
grading	1.35	Low collinearity
necrosis	1.16	Low collinearity
TILs in the tumor	1.17	Low collinearity
TILs in the VTT	1.15	Low collinearity
STING in the VTT	1.23	Low collinearity
